# The Hidden Geometries of the *Arabidopsis thaliana* Epidermis

**DOI:** 10.1371/journal.pone.0043546

**Published:** 2012-09-11

**Authors:** Lee Staff, Patricia Hurd, Lara Reale, Cathal Seoighe, Alyn Rockwood, Chris Gehring

**Affiliations:** 1 Geometric Modeling and Scientific Visualization Centre, King Abdullah University of Science and Technology, Thuwal, Kingdom of Saudi Arabia; 2 Department of Applied Biology, University of Perugia, Perugia, Italy; 3 School of Mathematics, Statistics and Applied Mathematics, National University of Ireland, Galway, Ireland; 4 Division of Chemistry, Life Science and Engineering, King Abdullah University of Science and Technology, Thuwal, Kingdom of Saudi Arabia; UMass, United States of America

## Abstract

The quest for the discovery of mathematical principles that underlie biological phenomena is ancient and ongoing. We present a geometric analysis of the complex interdigitated pavement cells in the *Arabidopsis thaliana* (Col.) adaxial epidermis with a view to discovering some geometric characteristics that may govern the formation of this tissue. More than 2,400 pavement cells from 10, 17 and 24 day old leaves were analyzed. These interdigitated cells revealed a number of geometric properties that remained constant across the three age groups. In particular, the number of digits per cell rarely exceeded 15, irrespective of cell area. Digit numbers per 100 µm^2^ cell area reduce with age and as cell area increases, suggesting early developmental programming of digits. Cell shape proportions as defined by length∶width ratios were highly conserved over time independent of the size and, interestingly, both the mean and the medians were close to the golden ratio 1.618034. With maturity, the cell area∶perimeter ratios increased from a mean of 2.0 to 2.4. Shape properties as defined by the medial axis transform (MAT) were calculated and revealed that branch points along the MAT typically comprise one large and two small angles. These showed consistency across the developmental stages considered here at 140° (± 5°) for the largest angles and 110° (± 5°) for the smaller angles. Voronoi diagram analyses of stomatal center coordinates revealed that giant pavement cells (≥500 µm^2^) tend to be arranged along Voronoi boundaries suggesting that they could function as a scaffold of the epidermis. In addition, we propose that pavement cells have a role in spacing and positioning of the stomata in the growing leaf and that they do so by growing within the limits of a set of ‘geometrical rules’.

## Introduction

**Figure 1:**
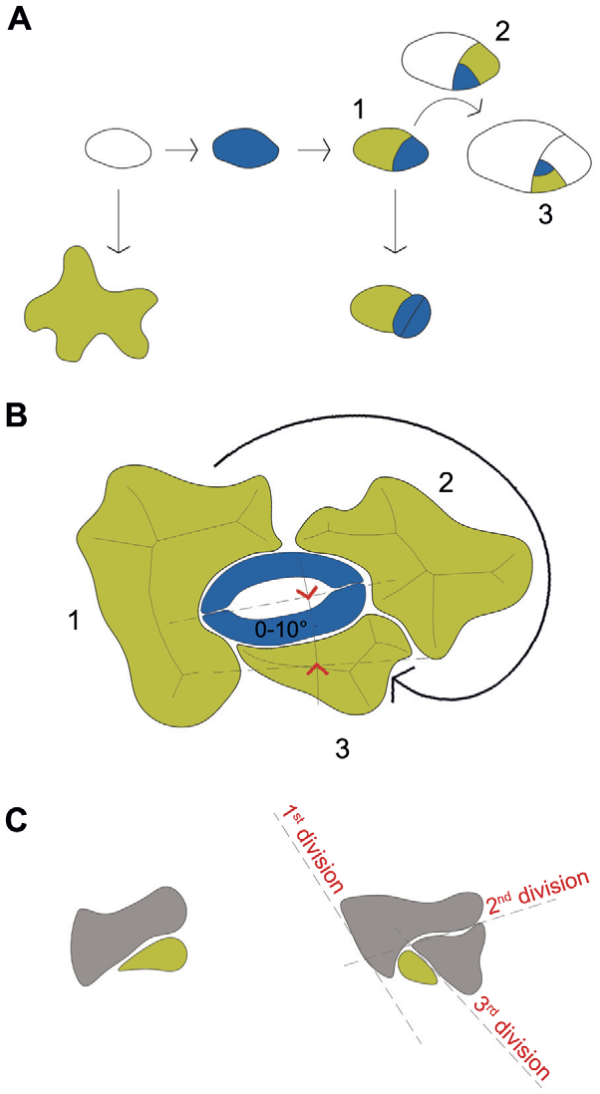
Illustrations of typical *Arabidopsis* cell division and differentiation. A Diagram of cell division in *Arabidopsis thaliana* showing meristemoids and stomata in blue, and pavement cells in green (or white). The cells labeled 1, 2 and 3 indicate oldest to youngest cells in that sequence of division. B Typical example of a pavement cell arrangement resulting from clockwise inward spiraling subdivision: first cell (1), second cell (2) and third cell (3). The axis of final pavement cell typically lies at 0°–10° to the axis of stomatal pore. The medial axis transform (MAT) lines of each cell are also shown. C This diagram represents a typical pavement cell in the early stages of division.

The *Arabidopsis thaliana* (Col.) epidermis consists of a single cell layer mostly composed of large, often polyploid, interdigitated cells with characteristic interlocking digits [1], also referred to as pavement cells. In interdigitated pavement cells, the digits (finger-like protrusions as illustrated in Figure 1A) have also been termed “lobes” [2] as well as “skeleton ends” [3]. Embedded into the pavement cells of the epidermis are specialized cells such as trichomes and stomatal guard cells and pores that allow for gas exchange with the parenchyma. Stomatal complex formation results from a final symmetric subdivision, forming the guard cells, preceded by a number of asymmetric cell divisions that usually spiral inwardly (Figure 1). This inward spiraling appears to be an ancient growth mode working in many plant tissues [4] and helps to maintain the advantageous one-cell spacing rule [5], [6], [7] that prevents stomatal complexes from crowding with immediate neighbors. Arguably, in the absence of a dedicated stomatal positioning mechanism, this spiraling development would give rise to a chaotic stomatal pattern. The interdigitated pavement cells surrounding the stomata fulfill the dual functions of protecting the internal tissues (preventing moisture loss, resisting pathogen invasion, holding internal material, and controlling temperature) and spacing out the more specialized cell types [8], [9]. Figure 1C illustrates how pavement cells can divide asymmetrically and how the daughter cells divide further. Whilst placement rules for cells of the initial division remain unresolved, it has been observed that the final division is oriented so that the angle between the axis of stomatal guard cells and the previous cell is usually between 0° and 10° [10].

The interdigitation of pavement cells is a complex morphogenetic process that requires a highly coordinated synthesis and operation of cortical microtubules and extensive remodeling of the cell wall [11]. It is likely that *A. thaliana* utilizes both position-dependent signals and lineage based patterns of division mechanisms to achieve an optimal stomatal distribution on the leaf surface [12].

Here we performed geometric analyses of the interdigitated pavement cells at different leaf ages with a view to extract shape patterns and rules of growth. We also performed Delaunay triangulation using stomatal center coordinates to deduce patterns of stomatal distribution across the leaf surface. The results from these analyses provide new insight into leaf architecture in general and, in particular, the impact of pavement cell geometry on stomatal development and spacing.

## Results

**Figure 2:**
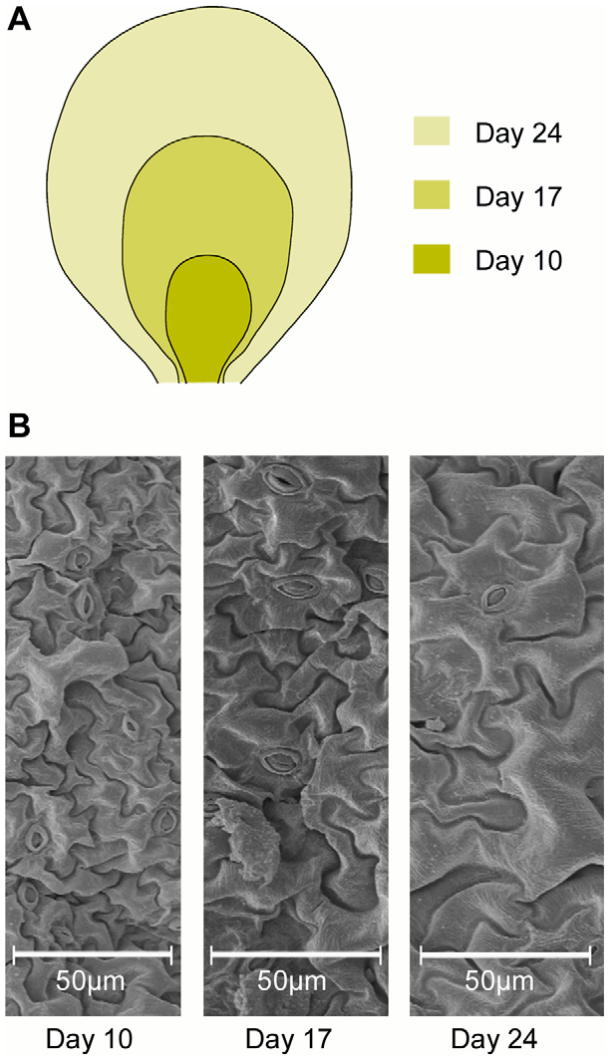
Growth stages of *Arabidopsis thaliana* (Col.) leaf. A Scanning Electron Microscope (SEM) images were taken from three different leaf ages, 10, 17 and 24 days after initial appearance of leaf. Day 10 leaf sizes ranged from 63 to 88 mm^2^ (average 75 mm^2^); day 17 leaf sizes ranged from 189 to 263 mm^2^ (average 237 mm^2^) and day 24 leaf sizes ranged from 559 to 809 mm^2^ (average 710 mm^2^). B Sample SEM images of *Arabidopsis thaliana* epidermal cells from three different ages as shown.

Data presented here was extracted from seventeen Scanning Electron Microscope (SEM) images of mid-leaf samples, taken approximately halfway between the central vein and the leaf edge, from *Arabidopsis thaliana* (Col.) plants harvested at different growth stages. The first SEM images were taken at day 10 after initial leaf appearance and 791 cells were measured and analyzed. From the day 17 samples, 929 cells were processed. The mature leaf samples were taken at day 24 and 739 cells were processed (Figure 2). At all three stages we observed interdigitated pavement cells.

### Bounding rectangle and length∶width ratio of pavement cells

**Figure 3:**
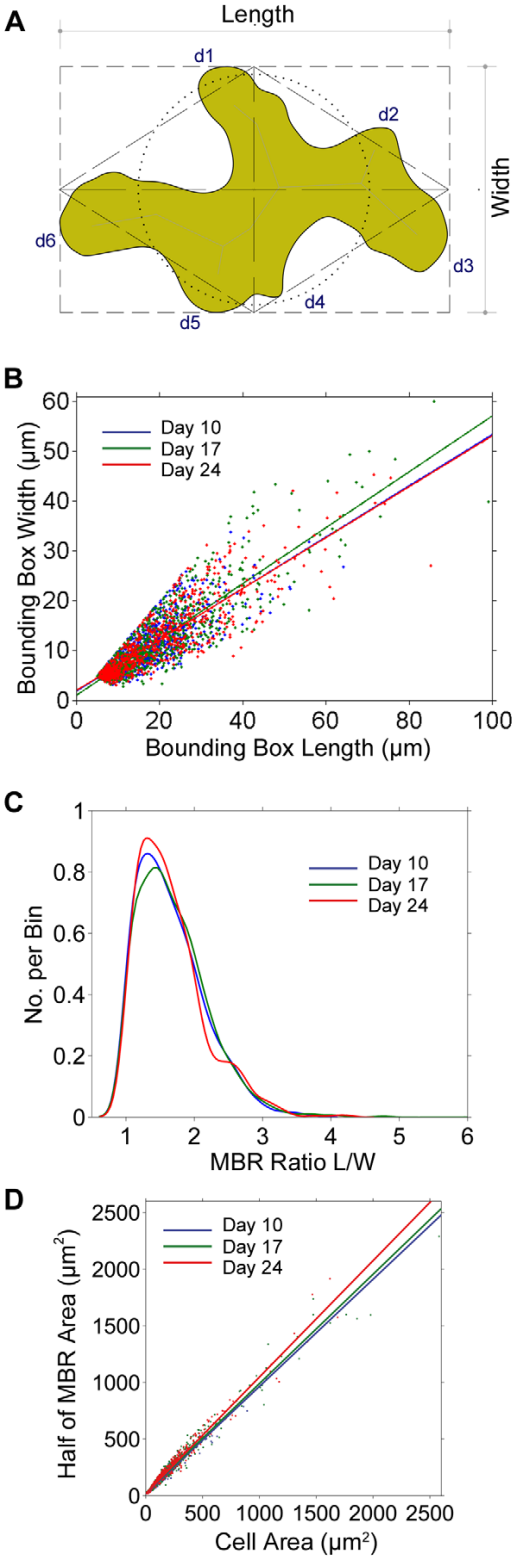
Cell shape as described by Minimum Bounding Rectangle (MBR). A Typical cell shape shown within its aligned MRB (short dashed line). The rhombus (long dashed line) represents half of the area of the MBR. Also shown, is the equivalent area circle (dotted line). B This graph plots MBR Length to MBR Width for each age group (days 10, 17 and 24). Mean length∶width ratios were 1.62 (95% CI 1.58–1.65), 1.65 (95% CI 1.62–1.68), and 1.60 (95% CI 1.57–1.64) for days 10, 17, and 24 respectively. Note: the golden ratio is 1.618034. C KS Density plots of MBR length∶width ratios for each day. Length∶width ratios were clustered in the range 1 to 3. Values rarely exceeded 3, and none were found to be greater than 6. D Plots the cell areas against half of the MBR. Interestingly, the linear fits lie almost exactly in line with the *x = y* plot, i.e. cell area = half of MBR.

In our first characterization of the pavement cells, we fitted minimum bounding rectangles aligned along the maximum length of each cell (Figure 3A). When the minimum bounding rectangles (MBR) were measured and plotted (Figure 3B) the length∶width ratio averages were 1.66, 1.70 and 1.66 for days 10, 17, and 24 respectively. The median length∶width ratios were 1.62 (95% CI 1.58–1.65) at day 10; 1.65 (95% CI 1.62–1.68) at day 17; and 1.60 (95% CI 1.57–1.64) at day 24. It therefore appears that the MBR length∶width aspect ratio shows conservation across growth stages despite differences in average cell sizes (day 10 linear fit was 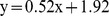; day 17 linear fit was 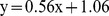; and day 24 linear fit was 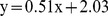). We note the closeness of these values to the golden ratio of 1.618034. In addition the length∶width ratio data shows sharp boundaries between one and three with only an insignificantly small number of cells having a ratio >3, and none with a ratio >6 (Figure 3C). The inferred density mode values were lower than the medians (1.30, 1.43 and 1.28 for day 10, 17 and 24), however, when day 24 cells with an area of ≥500 µm^2^ were isolated, the mode was 1.72 (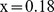). Remarkably, the ratio of the MBR to the total area is 2∶1 (Figure 3D), i.e. the cell area is almost always equivalent to half of the MBR area (see rhomboid in Figure 3A). In summary, several aspects of the shapes of the cells are invariant across development stages and cell size. These characteristics are typical for the cells in our sample that originate from the mid-leaf area whereas giant pavement cells, e.g. in the sepal, can greatly exceed the maximum length∶width ratio seen in our samples.

### The area∶perimeter ratio of pavement cells

**Figure 4:**
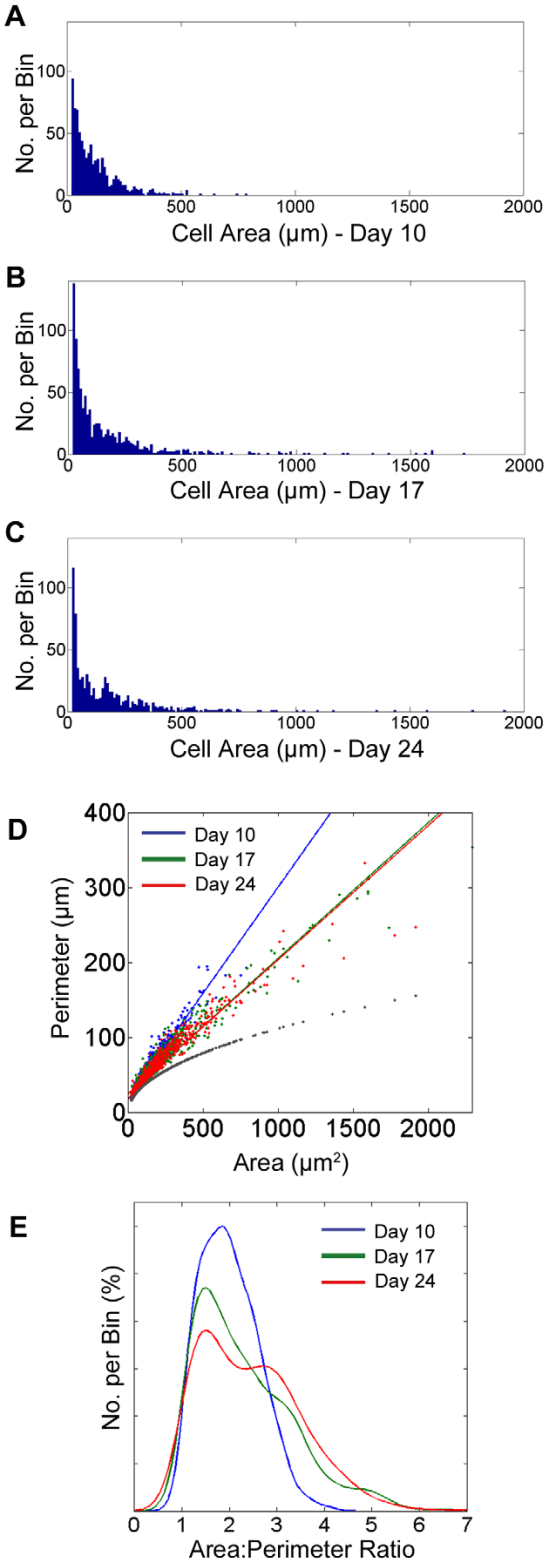
Cell Area analyses and Area versus Perimeter ratios. A, B & C Show pavement cell area histograms for day 10, day 17 and day 24 from top to bottom. Day 10 pavement cell areas range from 20 to 786 µm^2^; average cell area = 120 µm^2^; median cell area = 102 µm^2^. Day 17 pavement cell areas range from 20 to 2289 µm^2^; average cell area = 165 µm^2^; median cell area = 119 µm^2^. Day 24 pavement cell areas range from 20 to 1914 µm^2^; average cell area = 178 µm^2^; median cell area = 138 µm^2^. D Shows a plot of cell area against cell perimeter for each day 10, 17 and 24. The grey points show the equivalent circle for day 24 data where area is the same and perimeter is found by 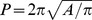. E KS Density plot of area∶perimeter ratio for days 10,17 and 24. The medians of the area∶perimeter ratios are 1.97 (95% CI 1.93–2.01), 2.23 (95% CI 2.16–2.30) and 2.38 (95% CI 2.30–2.38) at days 10, 17 and 24 respectively.

While few pavement cells were found to have an area larger than 500 µm^2^, the cell sizes varied considerably. At day 10, cell size ranged from 20 µm^2^ to 786 µm^2^, whilst the average cell size was 120 µm^2^ (SD 105) and the median was 102 µm^2^. At day 17 the range was 20 µm^2^ to 2289 µm^2^, the average was 165 µm^2^ (SD 225) and the median was 119 µm^2^. At day 24 the range was 20 µm^2^ to 1914 µm^2^, the average was 178 µm^2^ (SD 212) and the median was 138 µm^2^ (Figure 4A–C).

When we compared ratios between cell area and perimeter in the three different age groups we found a similar relationship in the three age groups (Figures 4D & 4E). The linear fit equations are 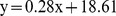 at day 10, 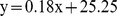 at day 17 and 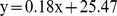 at day 24. As expected, the area∶perimeter scatter plots show that with maturation, we see a greater number of cells of larger area and perimeter. These larger cells, >500 µm^2^, are discussed further below. The maximum ratio increases from 4 (day 10) to 7 (day 17 & 24). Histograms and kernel density estimate (KS) plots reveal a distinct distribution peak around 1.5 and, qualitatively, suggest a second peak around 3 by day 24 (Figure 4E). Statistically, however, bimodality was not confirmed using the Hartigan's dip test. The medians of the area∶perimeter ratios were 1.97 (95% CI 1.93–2.01); 2.23 (95% CI 2.16–2.38) and 2.38 (95% CI 2.30–2.38) at days 10, 17 and 24 respectively. The equivalent circle fit for the same data (Figure 4D, grey points) shows a much sharper decrease in perimeter relative to the same area.

### Medial Axis Transform of pavement cells

**Figure 5:**
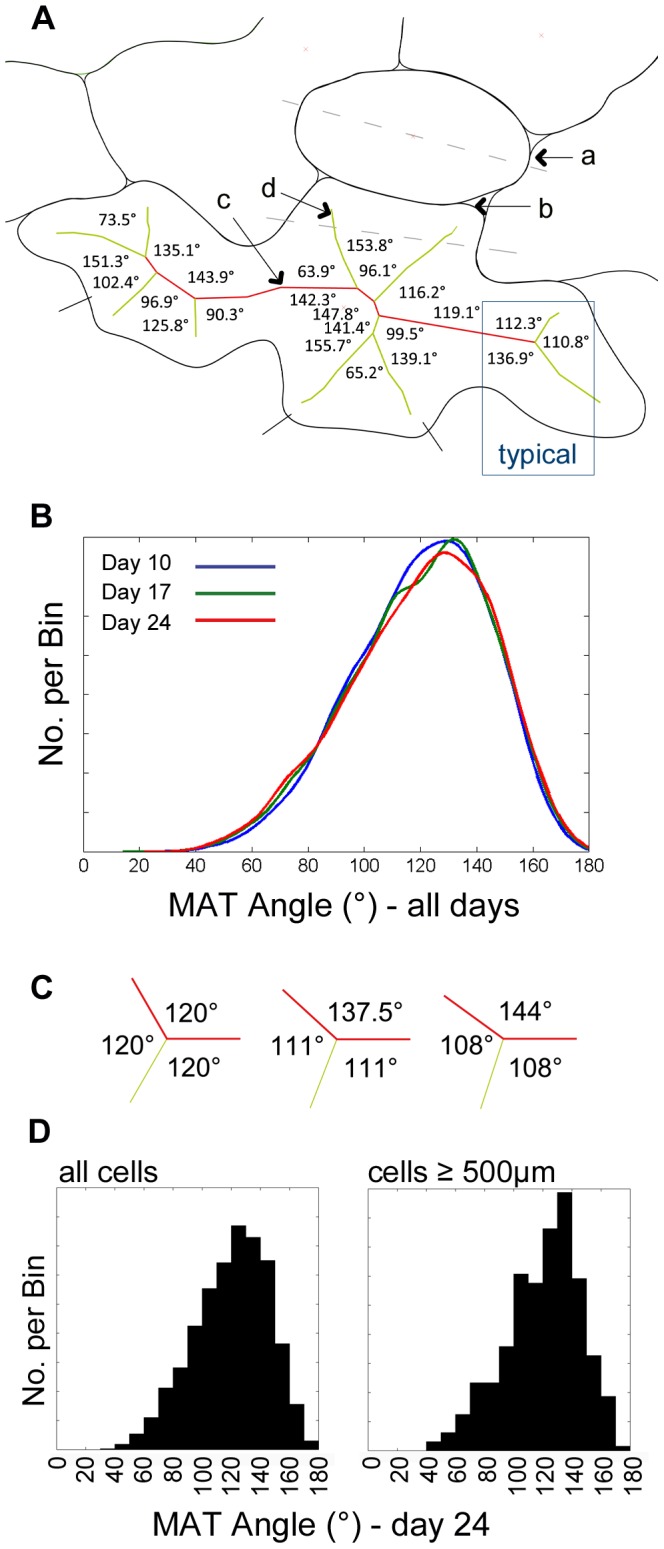
Medial Axis Transform (MAT) angles and distribution frequency. A Each cell outline was exported from AutoCAD into MATLAB using AutoLISP script. Here is the typical data extracted showing (a) stomatal pore axis; (b) cell boundary; (c) MAT internal segment lengths (red); and (d) external segment lengths (green). MAT angle measurements are also shown. B Graph shows the distribution curves of all angles along MAT at branch points for day 10 (blue), day 17 (green) and day 24 (red). The peaks all fall in the range 125–135°. C Possible angle subdivisions of 360°, e.g. golden angle plus two equal smaller angles would be 137.5°+111.25°+111.25°. Typical angle groupings found in our data set often exhibited such values, e.g. 144°+108°+108°. It is worth noting that the ratios of successive Fibonacci numbers converge on the golden ratio (137.5°) as *n* approaches infinity in the equation 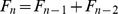, where 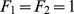. D Histograms of MAT angles for all cells (left) and only cells of area ≥500 µm^2^ (right) for Day 24. Generally the peak is around 130°, but when the giant cells are isolated, the increased frequency is seen around 100–110° and 130–140°.

The Medial Axis Transform (MAT) is a shape descriptor tool particularly apt for the description of closed curvilinear forms. It allows for the quantification of segment length and branch point angles [13]. The MATs of individual cells were extracted and both the digit (or lobe [2]) length segments and the internal segment lengths between digit branch points were analyzed (Figure 5A). Histograms of distribution frequency of segment lengths show only small variation across age groups (not shown). Both digit length and internal segment length distribution curves flattened out with maturity, which is an indication of cell elongation during differentiation towards maturity. Digit length maxima increased as follows: 22 µm at day 10, 29 µm at day 17 and 38 µm at day 24 and the majority of lengths (95%) fell in to the range of 0 µm–15 µm. The internal MAT segment length rarely (<5%) exceeded 15 µm in each age group suggesting that as the pavement cells grow, new digits will appear along the MAT at intervals ≤15 µm.

Histograms of all angles along the MAT at digit branch points show a non-normal, skewed distribution peaking at between 125° and 135° in each age group (day 10, 17 & 24) and no angle exceeded 180°. This distribution is highly conserved across the age groups (Figure 5B). All three angles were measured at each branch point and hence the average angle was 120°. The median of all angles at digit branch points was 121° in all three leaf sample groups. However, at each branch point, there is almost always one large angle and two smaller angles of similar magnitude (Figure 5A, see ‘typical’ example). In a further calculation we grouped the angles into two subsets. In the first subset, only the largest angles are computed and both the means and medians were similar and consistent across age. The means were 143.6°, 144.3° and 144.6° for day 10, 17 and 24 respectively whilst the medians were 143.3° (95% CI 142.9–143.7); 144.0° (95% CI 143.5–144.5); and 144.4° (95% CI 143.9–144.9). Furthermore, the modes, from an inferred density, were 140.4 (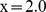), 137.6 (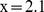) and 144.5 (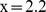) for day 10, 17 and 24 respectively. In the second subset, we considered the two smaller angles at branch points. The mean for all ages was 108° and the medians at the respective ages were 109.1° (95% CI 108.6–109.6), 108.8° (95% CI 108.2–109.4) and 108.7° (95% CI 108.0–109.3). The actual samples rarely exhibit perfect subdivisions and Figure 5C shows examples of idealized angle distribution of 360° with one large angle and two equal smaller angles. When cells of area >500 µm^2^ were analyzed separately (Figure 5D, right), we found that angles between 100–110° and 130–140° were more prevalent compared to the histogram including all cells (Figure 5D, left) and the modes, from an inferred density, were 135.7 (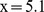), 139.3 (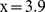) and 134.3 (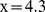) for day 10, 17 and 24 respectively.

### Digit numbers per cell area

**Figure 6:**
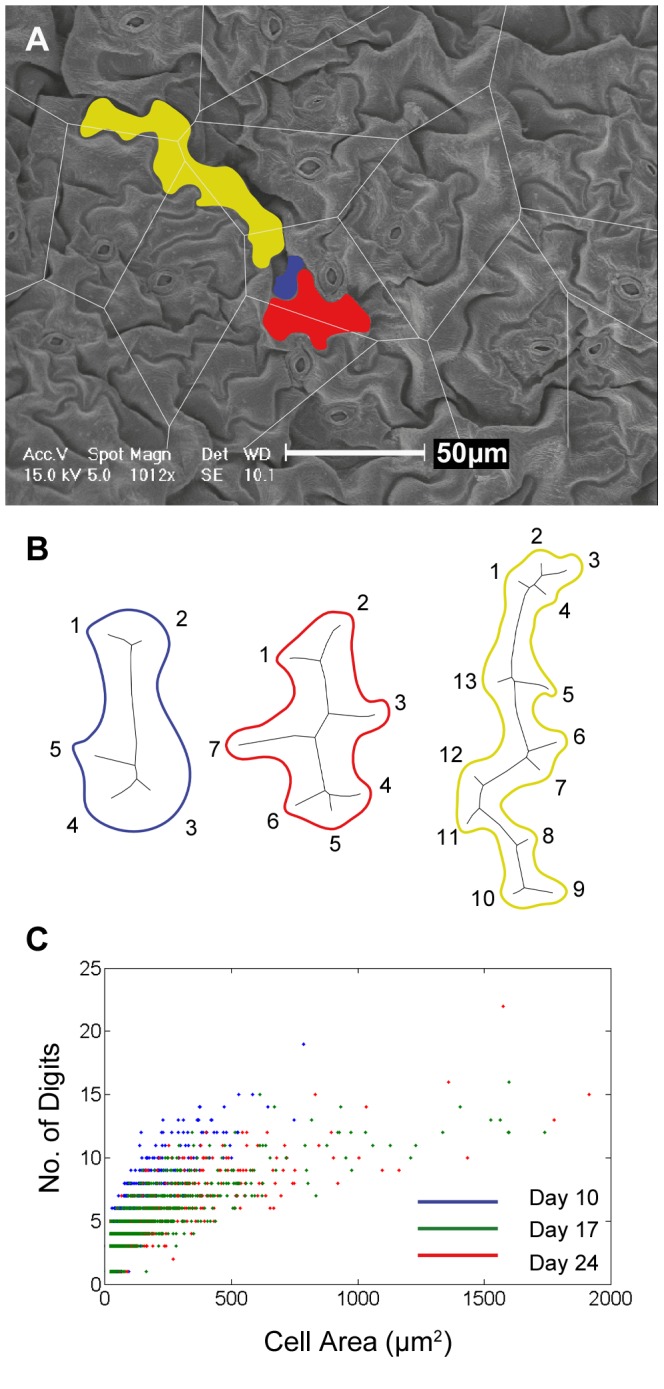
SEM image from day 24 data set: cell shapes and digit numbers per cell area. A SEM image taken from the day 24 series. Shown overlaid (grey lines) is the Voronoi diagram calculated from stomatal centers. Three cells are selected to represent cells of different sizes. B Boundaries of the cells identified in A: blue cell has area of 88 µm^2^ and 5 digits; red cell has area of 519 µm^2^ and 7 digits; green cell has area of 1032 µm^2^ and 13 digits. The ratio of digits per 100 µm^2^ is 5.6, 1.3 and 1.2 respectively for the blue, red and green cell shown here. For the purposes of shape comparison, the three cells have been re-scaled so that each has roughly the same area. C Scatter plot showing number of digits versus cell area, for the three different age groups. The range of digit numbers is from 1 to 22 and rarely exceeds 15 irrespective of cell size. Also, we see in the younger samples that the digit to area ratio is higher than in the mature samples. Mean no of digits per 100 µm^2^ for all cells: 6.7 (SD 3.6), 5.6 (SD 3.9) and 5.1 (SD 3.7) for day 10, 17 & 24 respectively. Mean number of digits per 100 µm^2^ for cells ≥500 µm^2^: 2.3 (SD 0.3), 1.3 (SD 0.4) and 1.3 (SD 0.4) for day 10, 17 & 24 respectively. Note: the data shown here in C is based on MAT data pruned to remove any external segment <0.55 µm.

Since cell sizes varied considerably (from <20 µm^2^ to 2289 µm^2^), we were interested to see whether the ratio of digit number to cell size also varied or whether it remained constant. Three examples of cells of different sizes taken from a day 24 image are shown (Figure 6A) and rescaled to visually demonstrate shape differences (Figure 6B). To understand the digit per area ratios, we plotted cell area against the number of digits for all cells of all growth stages (Figure 6C). Across all cells, the number of digits ranged from 1 to 22 but rarely exceeded 15, irrespective of cell size. We found the mean number of digits per 100 µm^2^ for all cells to be 6.7 (SD 3.6), 5.6 (SD 3.9) and 5.1 (SD 3.7) for day 10, 17 & 24 respectively. When the cells were divided into two groups according to size (<500 µm^2^ and ≥500 µm^2^), we noted that cells ≥500 µm^2^ have a considerably smaller mean number of digits per 100 µm^2^, i.e. 2.3 (SD 0.3), 1.3 (SD 0.4) and 1.3 (SD 0.4) for day 10, 17 & 24 respectively. Hence, we see that in the samples from younger leaves, the digit to area ratio is higher than in the mature samples, suggesting that the digits are formed early on in the development of a pavement cell and maintained throughout cell differentiation.

### The distribution of stomata

**Figure 7:**
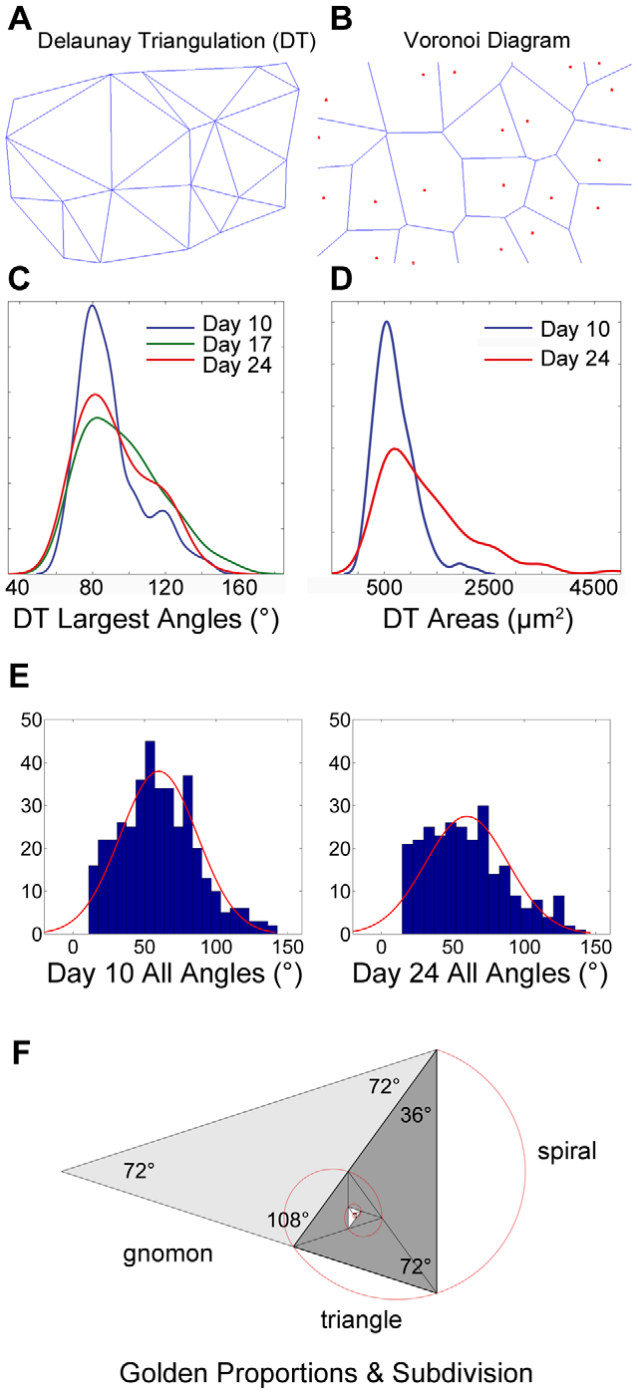
Delaunay Triangulation (DT) and Voronoi analysis based on stomatal centers; KS density plots of largest DT angles and DT areas. A Delaunay triangulation was conducted on each SEM image using the centroids of the stomata, i.e. stomatal pore and surrounding guard cells. B Voronoi diagram using centroids of stomata (centroids shown red) as seen in SEM images. C KS density distribution of the largest angles of the Delaunay triangulation: day 10 (blue), day 17 (green), and day 24 (red). Peaks are consistently around 80°–90°. D KS density distribution plots of DT areas for day 10 (blue) and day 24 (red). Peaks are consistent around 500 µm^2^. E Histogram with normal distribution fit overlay for all DT angles at day 10 (left) and day 24 (right). F Diagram exemplifies a golden spiral overlaying the golden triangle and golden gnomon.

In order to describe the distribution of stomata, we performed Delaunay triangulation on the stomatal pore center coordinates extracted from SEM images (Figure 7A). Delaunay triangulation ensures that the circumcircle associated with each triangle contains no other point in its interior [14]. We also constructed Voronoi diagrams (Figure 7B), the dual of the Delaunay triangulation, which is formed by connecting the centers of DT circumcircles. The median of all angles was 58°. When the largest angles were isolated (n = 130 for day 10; n = 130 for day 17; n = 87 for day 24), the histogram showed a markedly skewed distribution with greatest number of angles falling into the 80°–90° bin (Figure 7C). Medians were 87.2° (95% CI 84.2–90.7); 95.2° (95% CI 91.3–99.3); and 91.5° (95% CI 86.8–96.5) for day 10, 17 and 24 respectively. The means of the smallest angles were all between 40° and 45°. The peak of the angle distribution signature is common to the samples at the three ages investigated.

When the lengths of the sides of the Delaunay triangles of the day 10 data set (n = 227) were plotted a peak emerged at 35 µm–45 µm (not shown). At later stages (day 17, n = 229 and 24, n = 168), the peaks were at 40 µm–50 µm. The range at day 10 was 12 µm–88 µm, at day 17 it was 12 µm–160 µm and at day 24 the range consolidated at 18 µm–122 µm. A comparison of the areas of the stomatal triangles at day 17 and day 24 show that although with growth and maturation larger DT triangles occur, i.e. upper range increases from 2300 to 4800 µm^2^ over time, triangle areas for the peaks at both days are at around 500 µm^2^ (Figure 7D). The median stomatal DT area at day 10 is 671 µm^2^ and 1040 µm^2^ at day 24.

**Figure 8:**
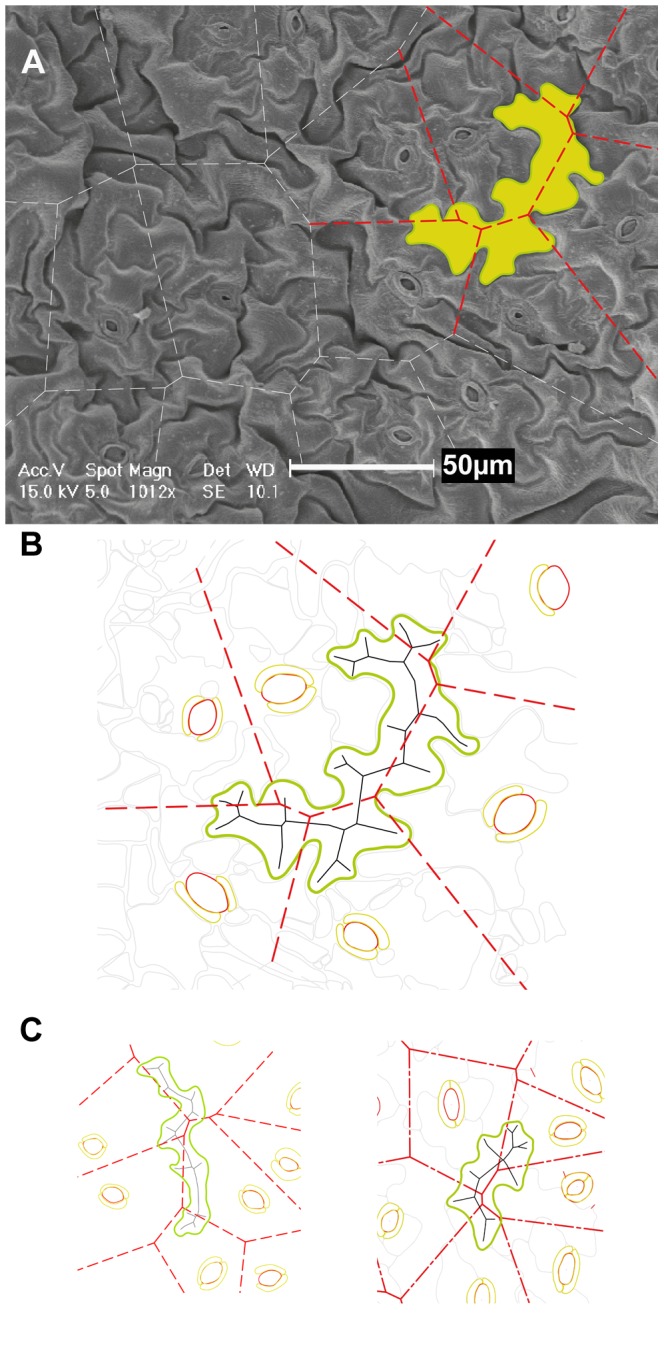
SEM image with giant pavement cell highlighted. A SEM image taken from the day 24 series with a giant cell outlined in green. B Giant pavement cell as identified in A: cell boundary (green line); medial axis transform (MAT; black lines); Voronoi cell boundaries (red dashed lines) calculated using centroid of stomatal complex as input coordinates; stomatal pores and guard cells (shown in red and yellow). Cell area = 1575 µm^2^, cell perimeter = 333 µm; area∶perimeter ratio = 4.7; average MAT angle is 139.4° for the largest angles and 110.3° for the smaller two of three angles. C Example (left), from day 24 data, of giant cell sitting along the Voronoi boundaries. Cell area = 1032; cell perimeter = 242; area∶perimeter ratio = 4.3; length∶width ratio = 3.2. Example (right) from a young leaf.

When Voronoi diagrams were computed and overlaid on the SEM images (Figure 8) it was observed that, particularly in the day 24 samples, the MAT of a number of the larger pavement cells coincided with the Voronoi cell boundaries. These cells also tend not to be immediately adjacent to stomata. In addition, these cells have area∶perimeter ratios greater than 3. It appears that these giant cells, i.e. cells with areas significantly greater than 500 µm^2^ and with length∶width ratios greater than 3, that trace the Voronoi boundaries could conceivably provide an overall scaffold-like structure that may also have an important role in the spacing of stomata.

## Discussion

Understanding cell patterning in general and in the plant leaf epidermis in particular continues to be of interest to cell biologists and geneticists. Cell distribution and patterning is non-random and is determined by a number of different factors including genetic pre-patterning mechanisms and pre-determined cell-fates, inhibitory signals that prevent neighbor cells from taking the same developmental fate, the position of the cells in the leaf as well as environmental conditions that impact on cell division, orientation and differentiation as well as cell-to-cell signaling and interactions [2], [9], [15], [16]. A number of extensive contributions have addressed cell patterning with particular emphasis on stomatal pores and guard cells [17], [18], [19]. However, little work to-date has explained the morphology of the so-called non-specialized pavement cell. Neither have their complex shapes been fully analyzed or interpreted.

Plant cells grow by increases in volume and cell wall surface area. The mature morphology of a plant cell is a result of the differential rates of expansion of neighboring zones of the cell [20], [21], a process that is particularly complex in the case of interdigitated cells [11]. This process also necessitates the coordinated action of filamentous actin arrays and actin-binding proteins. Although pavement cells appear irregular in shape, we were interested to see if any shape features were consistent or scale invariant. Firstly, we noted that the length∶width ratios in the maximally aligned bounding rectangle, a measure of the overall proportion of the cells, only rarely exceed 3 and median values were consistently around 1.6 in all three samples (10, 17 and 24 days). It is noteworthy that the mean values are very close to the golden ratio of phi (Π = 1.618034). In nature, many diverse structures exhibit the golden ratio and it has even been observed at the atomic scale in the magnetic resonance of spins in cobalt niobate crystals [22]. Phi has long been observed in the growth patterning of whole organisms such as in plant branching, spiral phyllotaxis [23], [24], and hence as an ordering principle in multicellular structures such as flowers and bracts, but it has never before been reported as a feature of single cells. One classic way that the golden ratio arises is through the successive subdivision of golden rectangles, or triangles, according to the golden ratio (see Figure 7F for idealized overlay of golden spiral and golden triangles). Stomatal complex formation requires (usually 3) asymmetric cell divisions of the meristemoid cell [25] followed by symmetric subdivisions forming the guard and companion cells. Asymmetric meristemoid cell divisions occur at angles reportedly close to 60° [26] leading to an inward spiral configuration which also assures that stomata are spaced apart by at least one cell. Is it possible that epidermal cell divisions (as shown in Figure 1) are geometrically characterized by golden proportions? On a macro level, phyllotaxis affords optimal access to light and optimal packing (economy of space) precisely because it is robust and relatively insensitive to perturbation [27]. We therefore hypothesize that a similar principle may be in place at the cellular level for similar reasons, i.e. optimized access to CO_2_ for the photosynthesizing tissue, and for cell packing and cohesion.

The average cell size increases during leaf maturation, however, the ratio of cell area to the area of the bounding rectangle remains almost 1∶2. Much like a jigsaw puzzle piece, the extents and ratio of cell remain fairly constant and the interlocking nature of the pieces require that the body only fill half of the extents (Figure 3D). Cells identified to have MBR length∶width ratio >3 may in fact belong to a special class of ‘giant’ cell and what looked like outliers, were in fact those cells that tend to lie along the Voronoi boundary (Figure 8A–C).

Pavement cell digit numbers ranged from 1 to 22 but rarely exceeded 15, irrespective of cell size, while the ratio of number of digits per cell area decreased significantly from the young to mature samples (Figure 6). Taken together, this suggests that the digit numbers and geometric properties of the pavement cell are determined early and maintained during leaf maturation, an interpretation consistent with recent findings [3].

The area∶perimeter ratio of cells, and their change during growth and development indicate the limits of interdigitation, including number and shape. We noted that the median area∶perimeter ratios increased with maturity from 2.0 at day 10 to 2.2 at day 17, and 2.4 at day 24. Future investigations will show whether the ratio reached at day 24 is an optimum under the conditions we tested, or if changes, e.g. in CO_2_ concentration, affect these proportions. We found that area versus perimeter values were remarkably similar at the different ages. The equivalent circle fit - the most efficient area∶perimeter ratio - for the same data (Figure 4D, grey points), shows a much sharper decrease in perimeter relative to the area. We therefore argue that perimeter efficiency optimization is not a constraint of the cell shaping process since the more costly, less perimeter efficient, structure may enable the spacing of specialized cells. Shapes with a high degree of area∶perimeter efficiency, such as the circle, would show a significantly flatter curve, i.e. as the area increases, the perimeter would increase at an ever more decreasing relative rate. It will be interesting to learn if environmental factors such as light intensity and quality, nutrient and water availability will affect area∶perimeter efficiency and, if so, how.

The analysis of cell MATs was aimed at finding geometric patterns that underlie cell growth directions, digit length and main cell axis characteristics during elongation growth in Arabidopsis pavement cells. Plant cell elongation growth in turn is defined as a cell extension growth mode that is not based on cell division (e.g. [20]). The interdigitated cells acquire their shapes as a result of differential cell elongation. The most remarkable feature is the skewed distribution of all angles along the MAT at digit branch points (Figure 5B) peaking at between 125° and 135°. Cell elongation patterns are best characterized by considering the set of largest angles along the medial axis. In turn, the smaller angles are representative of the branching pattern from the main medial axis (Figure 5A & C). Therefore we grouped the MAT angles according to these two subsets and found that they were conserved across the three developmental stages examined for both the larger and the smaller angles (Figure 5B). We therefore propose an elongation growth rule where changes in growth direction appear at an angle of 140° (± 5°). Similarly, we propose that digits will branch off from the main axis at an angle of 110° (± 5°). Such a growth mode is diagrammatically generalized in Figure 5C where 360° are divided into three principle angles, e.g. 144°, 108° and 108°. It worth noting here that the ratio of two consecutive numbers in the Fibonacci series (1, 1, 2, 3, 5, 8, 13, 21, 34, 55, 89, 144, ….) converges on φ (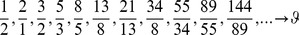)(see [28]). The ratio of alternate pairs as a proportion of 360° converges on the golden angle of 137.5°, i.e. 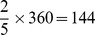 and 
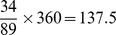
. This leaves a remaining angle of 222.5° or two smaller angles of 111.25°, i.e. divisions similar to those in the data presented here.

Stomatal distribution as described by Delaunay triangulation revealed that the subgroup of largest angles peaked at 80°–90° whereas the subgroup of smallest angles peaked at 40–45°. The equilateral triangle does not appear to be the most prevalent DT distribution and the day 24 data shows a range that is considerably broader with greater frequency of angles between 30° and 80°. As discussed earlier, to achieve optimal stomatal spacing within a robust and dynamic framework, it is conceivable that stomatal triangulation may push toward golden proportions, i.e. angles near to 36° and 72° and even 108° (Figure 7F). DT side length distributions show that the stomatal spacing remains remarkably regular during leaf growth, however, the smallest sides (12 µm–18 µm) at day 10 tend to disappear at later stages. Comparison of the areas of the stomatal triangles reveals that the majority of triangles have an area of between 200 µm^2^ and 1500 µm^2^ and that larger triangles, ≥1500 µm^2^, develop in the older samples when the leaf has fully expanded. The results showed that the median DT surface to pavement cell surface ratio increased from 16 at day 10 to 22 at day 24 and therefore suggests that increased spacing of stomata is a consequence of both cell elongation growth as well as an increase in cell numbers due to cell division.

Perhaps the most intriguing observation is the spatial orientation and identification of the giant pavement cells with surface areas between 500 µm^2^ and 2000 µm^2^. When the Voronoi diagram, constructed with stomata as centers, was overlaid on the scans, this subgroup of cells was noted often to lie approximately along the Voronoi boundaries (Figure 8). These cells are noticeably oversized and exhibit proportions longer and thinner than the majority of cells. So, do these giant cells play a role in the macro spacing of specialized cells, in particular the stomata? We speculate that the larger of the fully differentiated pavement cells are not clonally related to the stomatal complex, but have arisen independently from meristemoids and that these large interdigitated cells mainly serve in the spacing out of neighboring stomatal complexes. Giant cells of elongated proportions are also found in the sepal [29], however, in the convex shaped sepal they are not markedly interdigitated. It is conceivable that the shape characteristics of these giant cells could enable them to support groups of smaller cells in a structural, scaffold-like manner. Future work both in the wild-type and selected mutants, will continue to characterize the biological roles of these giant cells in epidermal growth in general and in the distribution of specialized cells in particular.

## Materials and Methods

### Sample preparation and scanning electron microscopy (SEM)

*Arabidopsis thaliana* (Col.) was grown under ambient light conditions. Leaves from the first rosette at three different developmental stages (10, 17 and 24 days after leaf emergence) were collected and fixed in 3% (w/v) glutaraldehyde in 0.075 M phosphate buffer, pH 7.0, for 5 h. The specimens were then washed four times for 15 minutes each in 0.075 M phosphate buffer, pH 7.0 and post-fixed in 1% (w/v) OsO_4_. Post-fixation samples were dehydrated in increasing concentrations of ethanol and dried with CO_2_ in a critical point drying (CPDA Jumbo, Polaron Equipment Limited, Walgord, England). The samples were coated with gold in an E5000C-PS3 Sputter coater (Agar Aids Instruments, Essex England) and observed with a scanning electron microscope (Cambridge Stereoscan 90B, Cambridge, USA).

Data presented here was extracted from seventeen SEM images: 6 at day 10, 5 at day 17 and 6 at day 24. The total number of cells that were measured and analyzed was 791 from day 10, 929 from day 17, and 739 from day 24 (Figure 2).

### Image processing and shape analyses

SEM images were imported into AutoCAD (Autodesk modeling software, 2011, www.autodesk.com). Image processing required acquisition of closed loop polylines defining the cell boundaries. Since this was not achievable with automated edge detection methods, edges were identified by eye and manually traced in AutoCAD using fit point splines.

For efficient processing of large data sets, we converted the splines to polylines (with subdivision set to 5) and exported the x,y coordinates of each cell boundary to MATLAB (R2011b, www.mathworks.com) using an AutoLISP script. In MATLAB, we used scripts to calculate cell sizes and proportions, minimum bounding rectangles of cells, and the medial axis transform (MAT) of cells [13]. The MAT of a shape is represented by a set of lines located at the midpoints of the boundaries of individual cells resulting in a skeletal representation of the overall shape that can be used to reconstruct the entire shape (Figure 5A). The initial MAT calculations resulted in a list of very small segments between branch points. We therefore developed a script to recursively traverse all the ‘neighbor’ line segments between branch points to connect them, i.e. internal segment. At this point, imperceptibly small digit segments (≤0.55 µm) were pruned off the MATs. The pruning parameters can be varied in the program. Similarly, the algorithm was used to connect digit segments. The scripts then calculated the angles at the branch points where the digit segments intersected the internal segments. The R Project for Statistical Computing was also used for calculations and the modes were from an inferred density (default bandwidth = x).

Stomatal patterning was analyzed using Voronoi diagrams and Delaunay triangulation both of which are standard routines in MATLAB. Stomatal pore center *x*,*y* coordinates were used as input data (Figure 6–8).
